# Effects of HBeAg Status on cellular immune function of patients with Hepatitis B virus/Treponema Pallidum Co-infectio

**DOI:** 10.12669/pjms.37.7.4253

**Published:** 2021

**Authors:** Wei Luo, Hanxiao Xin, Pengyun Zhao, Shasha Jiang

**Affiliations:** 1 Wei Luo, Blood Transfusion Department, Baoding First Central Hospital, Baoding, Hebei, 071000, China; 2 Hanxiao Xin, Blood Transfusion Department, Baoding First Central Hospital, Baoding, Hebei, 071000, China; 3 Pengyun Zhao, Blood Transfusion Department, Baoding First Central Hospital, Baoding, Hebei, 071000, China; 4 Shasha Jiang, Department of Convalescent, Tianjin Rehabilitation and Recuperation Center of PLA Joint Logistics Support Force, Tianjin, 300191, China

**Keywords:** HBeAg, HBV, TP, Co-infection, Cellular immune function

## Abstract

**Objectives:**

This study was aimed at exploring the effects of hepatitis B envelope antigen (HBeAg) status on the cellular immune function of patients with hepatitis B virus/treponema pallidum (HBV/TP) co-infection.

**Methods:**

The clinical data of 79 patients with HBV/TP co-infection admitted to our hospital from January 2019 to January 2020 were retrospectively analyzed. These patients were divided into two groups according to the different HBeAg statuses before the treatment: 41 HBeAg+ patients were included in the HBeAg+ group, while 38 HBeAg- patients were included in the HBeAg- group. The levels of HBV-DNA, T lymphocyte subsets represented by NK cells and cytokines associated with T cells in the peripheral blood (PB) of the patients were compared between both groups.

**Results:**

The HBV-DNA levels in the HBeAg+ group were significantly higher than those in the HBeAg- group (*P* < 0.05). The levels of CD3^+^, CD4^+^, CD4^+^/CD8^+^ and natural killer (NK) cells in the HBeAg+ group were higher than those in the HBeAg- group (*P* < 0.05), while the levels of CD8^+^ cells were lower than those in the HBeAg- group (*P* < 0.05). Moreover, the levels of interferon-γ (IFN-γ), tumor necrosis factor (TNF-α), interleukin-2 (IL-2), interleukin-6 (IL-6), interleukin-17 (IL-17), transforming growth factor-β (TGF-β) in the HBeAg+ group were all significantly higher than those in the HBeAg- group (*P* < 0.05), but there was no significant difference in the levels of interleukin-4 (IL-4) and interleukin-10 (IL-10) between the HBeAg+ group and the HBeAg- group (*P* > 0.05).

**Conclusion:**

HBeAg+ can increase the HBV-DNA levels in the PB of patients with HBV/TP co-infection, in turn triggering the body to initiate cellular immunity, increasing the levels of T lymphocyte subsets, and promote the secretion of cytokines.

## INTRODUCTION

Chronic hepatitis B (CHB) is a transmissible disease that arise out of hepatitis B virus (HBV) infection.^1^ Syphilis is a chronic systemic sexually transmitted disease cause by *Treponema pallidum* (TP) infection.^2^ Since HBV and TP share the same bodily-fluid transmission routes, co-infection involving both viruses are very common. In an HBV/TP co-infection, the patient’s autoimmune system makes a series of immune responses to participate in the pathological process within the body, injuring and damaging liver cells. In this process, cellular immunity mediated by T lymphocyte subsets and relevant cytokines plays a key role.^3^ Studies have shown that HBV can produce inhibitory monocytes, initiate the regulation of NK cell differentiation, and induce NK cells to produce IL-10.^4^ Hepatitis B envelope antigen (HBeAg) is a viral protein released from liver cells into the blood during HBV replication by the pre-core regions and core regions of the virus genes. HBeAg+ indicates active replication of HBV.^5^ Relevant studies have shown that, pre-treatment HBeAg status can affect the long-term antiviral effect of patients with HIV/HBV co-infection.^6^ However, whether HBeAg status can affect the cellular immune function of patients with HBV/TP co-infection still needs further exploration. On this basis, the clinical data of 79 patients with HBV/TP co-infection were reviewed, after which the effects of different HBeAg statuses on the cellular immune function of patients with HBV/TP co-infection were explored and reported.

## METHODS

The clinical data of 79 patients with HBV/TP co-infection admitted to our hospital from January 2019 to January 2020 were retrospectively analyzed. These patients were divided into two groups according to their HBeAg statuses before the treatment: 41 HBeAg+ patients were included in the HBeAg+ group, while 38 HBeAg- patients were included in the HBeAg- group. There were 24 male and 17 female patients in the HBeAg+ group; these patients aged 18-69, averaging (39.72±6.18) years; their body mass indices (BMIs) fell between 15.48 kg/m^2^ and 23.22 kg/m^2^, averaging (18.60±2.55) kg/m^2^; their courses of disease lied between six months and 20 months, averaging (12.54±2.31) months; 22 patients had a family history of HBV infection and 10 had a family history of TP infection; types of HBV infection included: CHB and chronic asymptomatic HBV, n = 13 and 28 respectively ; types of TP infection included: primary syphilis, secondary syphilis, early latent syphilis, and advanced latent syphilis, n = 11, 10, 16, and four respectively. There were 22 male and 16 female patients in the HBeAg- group; these patients aged 18-71, averaging (40.15±6.32) years; their BMIs fell between 15.23 kg/m^2^ and 23.54 kg/m^2^, averaging (18.72±2.68) kg/m^2^; the courses of disease lasted for 5-18 months, averaging (12.07±2.19) months; 20 patients had a family history of HBV infection and nine had a family history of TP infection; types of HBV infection included: CHB and chronic asymptomatic HBV, n = 11 and 27 respectively; types of TP infection included: primary syphilis, secondary syphilis, early latent syphilis, and advanced latent syphilis, n = 10, 9, 15, and 4 respectively. Also, the general data of patients between both groups showed no statistical difference (*P* > 0.05).

### Ethical approval

The study was approved by the Institutional Ethics Committee of Baoding First Central Hospital, and written informed consent was obtained from all participants.

### Inclusion criteria

patients who met the diagnostic criteria for HBV infection^7^; patients who meet the diagnostic criteria for TP infection^8^; patients in HBeAg+ group having had at least six months of HBeAg+; patients who received neither anti-viral nor immunoregulatory therapy before the inclusion into the study; patients who had complete clinical data.

### Exclusion criteria

patients who also contracted hepatitis A, C, D, E viruses or other viruses; patients with liver injuries caused by alcohol and drug allergic poisoning; patients with autoimmune liver disease (AILD); patients with severe organ failures, including but not limited to the heart, lung, and kidney; patients with malignant tumors; patients who had received immunomodulatory therapy within the last 6 months; pregnant or lactating women; and patients with mental disorders, etc.

3 mL PB was sampled from the patients upon admission, and then centrifuged at 3000 r/min for 5 min to separate the serum for DNA extraction. The serum HBV-DNA levels were measured using a Roche LightCycler 480 II Real Time PCR System via a real-time quantitative polymerase chain reaction (RT-PCR). The kit selected was the Power SYBR Green PCR Master Mix kit manufactured by the US-based ABI. All operations were carried out in strict accordance with the instructions provided within the kit.

The levels of CD3^+^, CD4^+^, CD8^+^ T lymphocytes and natural killer (NK) cells in the PB were measured with monoclonal antibodies and Cytoflex flow cytometer (Beckman, USA). Analyses involving all recorded counts and statistics as well as CD4^+^/CD8^+^ calculations were carried out with FACSCanto. 3 mL PB was sampled from the patients and centrifuged at 3000r/min for five minutes to separate the serum. The levels of interferon-γ (IFN-γ), tumor necrosis factor (TNF-α), interleukin-2 (IL-2), interleukin-4 (IL-4), interleukin-6 (IL-6), interleukin-10 (IL-10), interleukin-17 (IL-17) and transforming growth factor-β (TGF-β) were detected via enzyme linked immunosorbent assay (ELISA) using a kit supplied by the US-based R&D. The instructions provided with the kit were strictly followed.

### Observation indicators

(1) Comparison of HBV-DNA levels between both groups; (2) Comparison of T lymphocyte subset levels in PB between both groups; and (3) Comparison of cytokine levels in PB between both groups.

### Statistical analysis

SPSS25.0 was used to process the statistics. The measurements were expressed in ““ and had their statistical significance determined using the *t* test. 0.05 was taken as the statistical significance threshold level.

## RESULTS

HBV-DNA levels in the HBeAg+ group were significantly higher than those in the HBeAg- group (*P* < 0.05). Refer to Table-I for more details.The levels of CD3^+^, CD4^+^ and CD4^+^/CD8^+^T lymphocytes as well as NK cells in the HBeAg+ group were higher than those in the HBeAg- group, while the levels of CD8^+^T lymphocytes being lower than those in the HBeAg- group. The comparison was statistically significant (*P* < 0.05). Refer to Table-II for more details.

**Table I:** Comparison of HBV-DNA Levels between both Groups (x¯±s ).

*Group*	*n*	*HBV-DNA (×10^6^ copies/mL)*
HBeAg+ group	41	38.92±6.13
HBeAg- group	38	20.77±3.41
t		16.086
P		0.000

**Table II:** Comparison of T Lymphocyte Subset Levels in PB between both Groups (x¯±s ).

*Group*	*n*	*CD3^+^ (%)*	*CD4^+^ (%)*	*CD8^+^ (%)*	*NK (%)*	*CD4^+^/CD8^+^*
HBeAg+ group	41	68.34±9.26	43.47±5.21	24.98±4.23	17.28±3.56	1.74±0.31
HBeAg- group	38	56.87±8.32	36.98±5.09	28.79±4.59	14.09±3.21	1.28±0.24
t		5.775	5.593	3.840	4.171	7.333
P		0.000	0.000	0.000	0.000	0.000

### Comparison of cytokine levels in PB between both groups

The levels of IFN-γ, TNF-α, IL-2, IL-6, IL-17 and TGF-β in the HBeAg+ group were significantly higher than those in the HBeAg- group, showing statistically significant differences (*P* < 0.05). On the contrary, there was no statistical significance in the IL-4 and IL-10 levels between the HBeAg+ group and the HBeAg- group (*P* > 0.05). See Table-III.

**Table IIIa:** Comparison of Cytokine Levels in PB between both Groups (x¯±s ).

*Group*	*n*	*IFN-γ (pg/mL)*	*TNF-α (pg/mL)*	*IL-2 (pg/mL)*	*IL-4 (pg/mL)*
HBeAg+ group	41	173.53±19.98	21.81±4.22	124.33±16.48	60.73±7.63
HBeAg- group	38	148.09±17.74	16.03±2.35	98.67±13.29	58.36±7.12
t		5.966	7.439	7.581	1.424
p		0.000	0.000	0.000	0.158

**Table IIIb:** Comparison of Cytokine Levels in PB between both Groups (x¯±s ).

*Group*	*n*	*IL-6 (pg/mL)*	*IL-17 (pg/mL)*	*IL-10 (pg/mL)*	*TGF-β (pg/mL)*
HBeAg+ group	41	59.37±8.82	234.26±24.78	114.31±11.22	320.54±36.72
HBeAg- group	38	51.42±7.18	178.37±20.09	112.35±10.18	286.54±27.16
t		4.373	10.959	0.811	4.649
P		0.000	0.000	0.420	0.000

## DISCUSSIONS

HBV belongs to the *Hepadnaviridae* family and is one of the most common and serious infectious diseases in the world. HBV infection may impair liver functions, and gradually develop into CHB, cirrhosis, liver cancer and other diseases.^9^ TP can enter the body through broken skin or mucous membranes and be transported to the whole body through the blood and lymphatic system to damage organs and tissues, ultimately resulting in functional loss and tissue necrosis.^10^ TP and HBV share similar infection routes. Therefore, the probability of co-infection between both is high. The co-infection, however, will accelerate the progression of cirrhosis in patients, which will cause serious harm to the physical and mental health of patients and increase the case fatality rate.^11^ HBeAg is a main protein in HBV’s core and is distributed in the blood and the whole body. HBeAg+ indicates that the virus is in an active replication state. Nevertheless, whether it affects and how it affects the cellular immune function of patients with HBV/TP co-infection are still unclear and need further study.

In this study, the serum HBV DNA levels in the HBeAg+ group were higher than those in the HBeAg- group, indicating that HBeAg+ promotes the activity of HBV virus replication. It is generally recognized that sensitive indicators reflecting HBV replication include persistent HBeAg positive and elevated levels of HBV DNA. When HBeAg+ is detected in blood, it indicates that the HBV replicates in large numbers and is highly infectious.^12^ Studies have shown that high HBV DNA load is an independent factor of liver fibrosis and an independent predictor of significant liver necrosis inflammation. Furthermore, liver histological stage and grade were higher in patients with high HBV DNA load by stratification.^13^ The results of this study indicating that when HBeAg is positive, the virus replicates in large numbers. The levels of CD3^+^, CD4^+^ and CD4^+^/CD8^+^ T lymphocytes and NK cells in the HBeAg+ group were higher than those in the HBeAg- group, while the levels of CD8^+^T lymphocytes were lower than those in the HBeAg- group, indicating that HBeAg+ can enhance the levels of T lymphocyte subsets. HBeAg+ indicates active replication of HBV virus and elevated HBV DNA levels. The body strengthens its autoimmune system in response to a large number of viruses. After HBV infects the human body, it does not directly cause hepatocyte injury. Instead, it indirectly damage hepatocytes by inducing the cellular immunity mediated by T lymphocytes and a part of the cytokines. After HBV/TP invades the human body, autoimmunity is initiated, causing T cells to target and attack cells containing the virus. CD3^+^ T lymphocytes are peripheral mature T cells. CD4^+^ and CD8^+^ are directly associated with cellular immune function.^14^ CD4^+^ T lymphocytes are a kind of helper T cells, and play an important role in immune responses to the virus: on the one hand, they can activate and kill harmful cells; on the other hand, they can help B cells to produce antibodies and boost immunity. CD8^+^ T lymphocytes are a kind of inhibitory and lethal T cells that can inhibit the synthesis and secretion of antibodies, and are mainly used for cell cleanup. Under normal circumstances, CD4^+^/CD8^+^ levels are kept at a certain proportion to jointly maintain the body’s autoimmune stability.^15^ Activated NK cells can synthesize and secrete a variety of cytokines for immune regulation and directly killing target cells. A study^16^ showed that, the CD3^+^, CD4^+^ and CD4^+^/CD8^+^ in patients infected with HBV decrease significantly, while CD8^+^ and IL-6 increase significantly. The study also reported that T cells and IL-6 levels are closely correlated to disease type and HBV-DNA load. The findings acquired herein are similar to the reports above, indicating that the CD3^+^, CD4^+^ and CD4^+^/CD8^+^T cell levels change in HBeAg+ patients with HBV/TP co-infection.

Moreover, in this study, the levels of most cytokines in the HBeAg+ group, such as IFN-γ, TNF-α, IL-2, IL-6, IL-17 and TGF-β, were higher than those in the HBeAg- group, while the IL-4 and IL-10 levels were equivalent to those in the HBeAg- group, indicating that HBeAg+ can promote patients with HBV/TP co-infection to secrete T lymphocyte-associated cytokines and involve it in the inflammatory reaction. Having contracted HBV/TP co-infection, the human body makes specific immune responses that trigger cellular immunity dominated by T lymphocytes. Cytokines are soluble small molecule polypeptides or glycoproteins produced and secreted by different immune cells, and are biological signals that play multiple functions in normal pathological and physiological processes.^17^ Having contracted HBV/TP, various immune cells may gather and be activated at the site of inflammation, inducing differentiation and proliferation of various cells, in turn releasing multiple cytokines to be involved in inflammatory and immune responses.^18^ As important mediators of inflammatory responses produced by hosts, cytokines are major players in T lymphocyte-mediated cellular immunity, and are involved in the occurrence and development of a variety of diseases. According to the variety of cytokines secreted, CD4^+^ T lymphocytes can be divided into T helper cell 1 (Th1), T helper cell 2 (Th2), T helper cell 17 (Th17) and regulatory T cells (Treg).^19^ Th1 cell-mediated cellular immunity is responsible for the body’s function in eliminating viruses. The cytokines secreted include IFN-γ, TNF-α and IL-2. Th2 cells kill virus-infected hepatocytes primarily through the cellular cytotoxicity of the immune cells, and mainly secrete such cytokines as IL-4 and IL-6. Th17 cells are helper T cells with proinflammatory effects, which demonstrate strong immunogenicity, and trigger the protective effects of immune response by immunoregulatory specific killing, blocking of signal pathway, etc.; their main effector cytokine is IL-17. Treg cells are regulatory T cells with anti-inflammatory effects; and their major immunity-triggering cytokines are IL-10 and TGF-β.^20^ When HBV replicates, the virus in the body proliferates, causing immune dysfunction. This intensifies the immune response, and stimulates the body to further enhance the response effects. At this point, immune cells secrete more cytokines, including TNF-α, IFN-γ, IL-6 and IL-17, to promote inflammatory reaction and enhance their immune function, at the expense of injuring and damaging liver cells and tissues. Previous studies have shown that, there are abnormal cytokine levels in the pathogenesis of HBV-associated nephritis in children. Therefore, combined monitoring of HBV-DNA and cytokines should be carried out during the treatment to dynamically evaluate the changes in condition and the therapeutic effects.^21^ The results of this study are close to the reports above, indicating that HBeAg+ triggers changes to the otherwise stable levels of T lymphocyte-associated cytokines in patients with HBV/TP co-infection.

## CONCLUSION

To sum up, HBeAg+ enhances HBV replication in patients with HBV/TP co-infection, and stimulates the body to initiate T lymphocyte-mediated cellular immunity, increase the levels of T lymphocyte subsets, and promote the release of a variety of cytokines to be involved in the body’s antiviral immune response.

### Authors’ Contributions:

**WL &** **HX:** Designed this study and prepared this manuscript, and are responsible and accountable for the accuracy or integrity of the work;

**PZ:** Collected and analyzed clinical data, significantly revised this manuscript.

## References

[1] Yin JL, Yang HQ (2019). In vivo immune response in patients with hepatitis B virus infection complicated with anxiety. China J Modern Med.

[2] Pastuszczak M, Kotnis-Gaska A, Jakubowicz B, Wojas-Pelc A (2019). Treponema pallidum-specific immune responses and autoimmunity in patients who remain serofast after treatment of syphilis. Postepy Dermatol Alergol.

[3] Wursthorn K, Wedemeyer H, Manns MP (2010). Managing HBV in patients with impaired immunity. Gut.

[4] Li H, Zhai N, Wang Z, Song H, Yang Y, Cui A, Li T, Wang G, Niu J, Crispe IN, Su L, Tu Z (2018). Regulatory NK cells mediated between immunosuppressive monocytes and dysfunctional T cells in chronic HBV infection. Gut.

[5] Boyd A, Kouame MG, Houghtaling L, Moh R, Gabillard D, Maylin S (2019). Hepatitis B virus activity in untreated hepatitis B e antigen-negative human immunodeficiency virus-hepatitis B virus co-infected patients from sub-Saharan Africa. Trans R Soc Trop Med Hyg.

[6] Nie Y, Lan Y, Li F (2018). Effects of HBeAg status on immune reconstitution in patients with HIV/HBV co-infection. Inter J Epidemiol Infect Dis.

[7] Chinese Society of Infectious Diseases, Chinese Medical Association;Chinese Society of Hepatology, Chinese Medical Association (2019). Zhonghua Gan Zang Bing Za Zhi.

[8] National Center for STD Control, China CDC, Department of Sexually Transmitted Diseases, Society of Dermatology, Chinese Medical Association, Sexually Transmitted Diseases Subcommittee, Society of Dermatologists, Chinese Medical Doctor Association. Guidelines for diagnosis and treatment of syphilis, gonorrhea, and genital tract chlamydia trachomatis (2020 version) (2020). Chinese J Dermatol.

[9] Fanning GC, Zoulim F, Hou J, Bertoletti A (2019). Therapeutic strategies for hepatitis B virus infection:towards a cure [published correction appears in Nat Rev Drug Discov 2020 Jan 8]. Nat Rev Drug Discov.

[10] Drago F, Javor S, Parodi A (2019). Relevance in biology and mechanisms of immune and treatment evasion of Treponema pallidum. G Ital Dermatol Venereol.

[11] Yang Q, Feng H, Abie Yihe (2020). Status quo of HIV/HBV, HCV and TP co-infection among Yi nationalities in a county in Liangshan Prefecture. Laboratory Med and Clinic.

[12] Chen HS, Wu JF, Su TH, Chen HL, Hsu HY, Xia NS (2019). Baseline Level of Hepatitis B Core Antibody Predicts Spontaneous Hepatitis B e Antigen (HBeAg) Seroconversion in HBeAg-Positive Children With a Normal Alanine Aminotransferase Level. Hepatology.

[13] Lee IC, Huang YH, Chan CC, Huo TI, Chu CJ, Lai CR (2011). Impact of body mass index and viral load on liver histology in hepatitis B e antigen-negative chronic hepatitis B. Clin Nutr.

[14] Sun ML, Song Y (2018). Correlation between CD4^+^T, CD8^+^T and CD4^+^/CD8^+^ and different constitutions of TCM in chronic hepatitis B. Genomics Appl Bio.

[15] Kokuina E, Breff-Fonseca MC, Villegas-Valverde CA, Mora-Diaz I (2019). Normal Values of T, B and NK Lymphocyte Subpopulations in Peripheral Blood of Healthy Cuban Adults. MEDICC Rev.

[16] Li YF, Yang XM, Yuan Y (2019). Expression and clinical significance of T cell and interleukin-6 levels in patients with hepatitis B virus infection. J Chengdu Med College.

[17] Boni C, Janssen HLA, Rossi M, Yoon SK, Vecchi A, Barili V (2019). Combined GS-4774 and Tenofovir Therapy Can Improve HBV-Specific T-Cell Responses in Patients with Chronic Hepatitis. Gastroenterology.

[18] Ma HX (2019). Effects of virus load on peripheral blood T lymphocytes, cytokines and liver function in patients with hepatitis B virus infection. Laboratory Med Clinic.

[19] Watanabe S, Yamada Y, Murakami H (2020). Expression of Th1/Th2 cell-related chemokine receptors on CD4+lymphocytes under physiological conditions. Int J Lab Hematol.

[20] Sifnaios E, Mastorakos G, Psarra K, Panagopoulos ND, Panoulis K, Vitoratos N (2019). Gestational Diabetes and T-cell (Th1/Th2/Th17/Treg) Immune Profile. In Vivo.

[21] Lei XY, Chen XX, Luo X (2020). Correlation between changes in T lymphocyte subsets and multiple cytokines and HBV-associated nephritis in children. Chinese J Applied Clin Pediatrics.

